# Supplementary material for: “Who Cares?”: The Acceptance of Decentralized Wastewater Systems in Regions without Water Problems
Source: Int J Environ Res Public Health. 2020 Dec 4;17(23):9060. doi: 10.3390/ijerph17239060 (PMC7730708; doi:10.3390/ijerph17239060)
Supplement: Supplementary file 1 [file ijerph-17-09060-s001.pdf]

Supplementary materials

Figure S1. Search terms that have been used for the selection of studies to be included in the literature review:

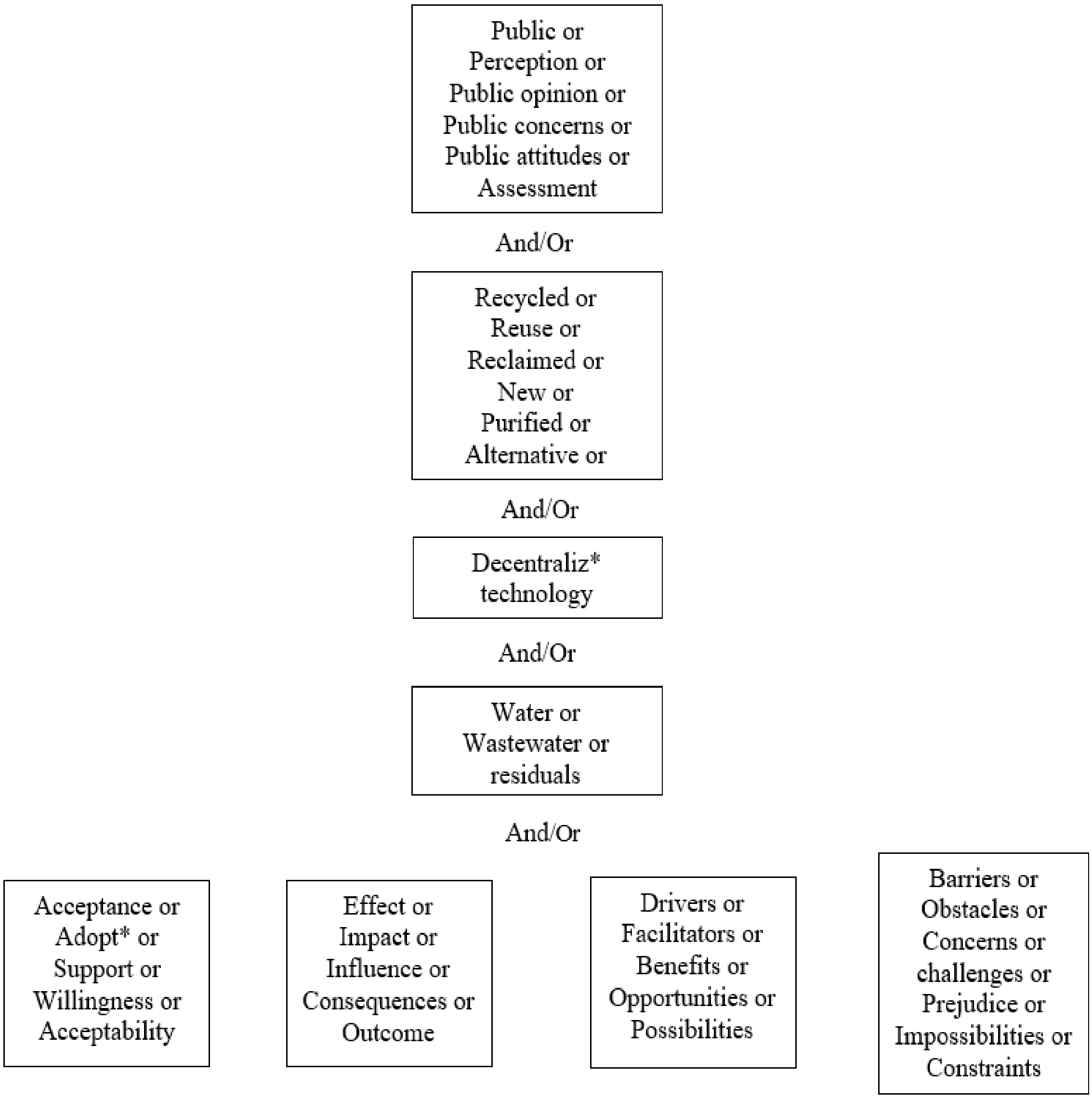

Figure S2. PRISMA diagram, which collects the different steps followed in the process for the final selection of studies included in the literature review.

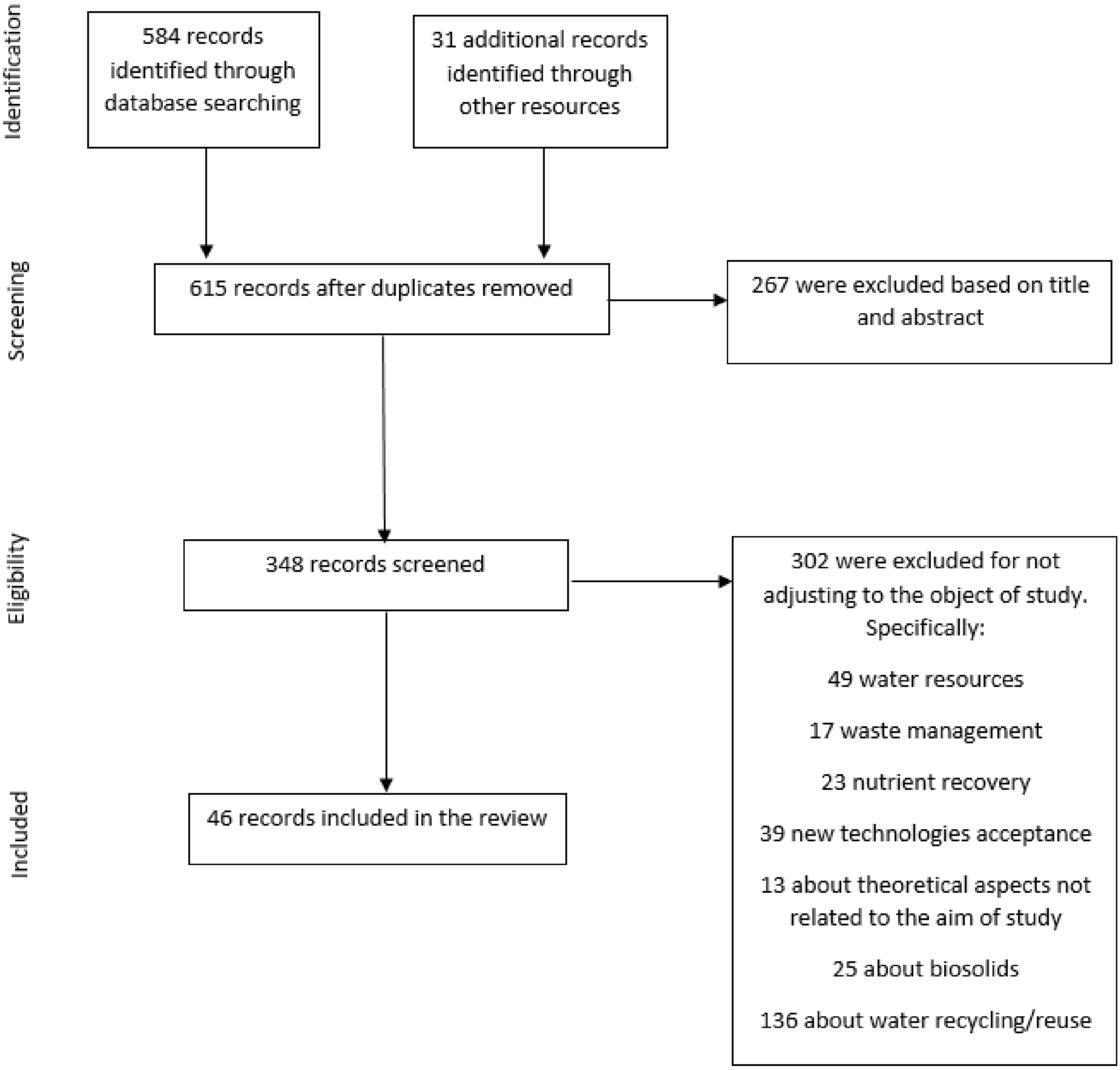

Table S1. References included in the literature review

1. Blume, S., & Winker, M. (2011). Three years of operation of the urine diversion system at GTZ headquarters in Germany: user opinions and maintenance challenges. *Water Science & Techonology, 64* (3), 579-586. doi: 10.2166/wst.2011.530
2. Branstrator J. M. S. (2014). *The barriers to adopting composting toilets into use in urban and suburban locations in the United States*. (Doctoral thesis). Indiana: Purdue University.
3. Chen R., Wang X. C. (2009). Cost–benefit evaluation of a decentralised water system for wastewater reuse and environmental protection. Water Science & Technology, https://doi.org/10.2166/wst.2009.156.
4. Chirisa I., Bandauko E., Matamanda A., Mandisvika G. (2017). Decentralized domestic wastewater systems in developing countries: the case study of Harare (Zimbabwe). *Applied Water Science*, https://doi.org/10.1007/s13201-016-0377-4.
5. Domènech L. (2011). Rethinking water management: From centralized to decentralised water supply and sanitation models. *Documents d'Anàlisi Geogràfica*, https://doi.org/10.5565/rev/dag.280.
6. Domènech L., Saurí D. (2010). Socio-technical transitions in water scarcity contexts: Public acceptance of greywater reuse technologies in the Metropolitan Area of Barcelona. Resources, *Conservation and Recycling*, https://doi.org/10.1016/j.resconrec.2010.07.001.
7. Domènech L., Vallès M. (2014). Local regulations on alternative water sources: greywater and rainwater use in the Metropolitan Region of Barcelona. *Investigaciones Geográficas*, http://dx.doi.org/10.14198/INGEO2014.61.06.
8. Farrelly M., Brown R. (2011). Rethinking urban water management: Experimentation as a way forward? *Global Environmental Change*, https://doi.org/10.1016/j.gloenvcha.2011.01.007.
9. Fielding K. S., Gardner J., Leviston Z., Price J. (2015). Comparing public perceptions of alternative water sources for potable use: The case of rainwater, stormwater, desalinated water, and recycled water. *Water Resources Management*, https://doi.org/10.1007/s11269-015-1072-1.
10. Gardiner A., Skoien P., Gardner T. (2008). Decentralised water supplies: South-East Queensland householders' experience and attitudes. *Water*, 35(1), 53–58.
11. Gikas P., Tchobanoglous G. (2009). The role of satellite and decentralised strategies in water resources management. *Journal of Environmental Management*, https://doi.org/10.1016/j.jenvman.2007.08.016.
12. Hegger D., van Vliet B., Spaargaren G., Vliet B. (2008). *Decentralised sanitation and reuse in Dutch Society: Social opportunities and risks: Final report for the EET-DESAR project*. The Netherlands: Wageningen University.
13. Ho G., Anda M. (2006). Centralized versus decentralised wastewater systems in an urban context: The sustainability dimension. 2nd IWA Leading-Edge Conference on Sustainability, (November 2004), 80–89.
14. Ignacio, J.J., Malenav, R.A., Pausta, C.M., Beltrán, A., Belo, L., Tanhueco, R.M., Promentilla, M.A., & Orbecido, A. (2019). A Perception Study of an Integrated Water System Project in a Water Scarce Community in the Philippines. *Water, 11*, 1593; doi:10.3390/w11081593

15. Jeffrey P. (2002). Public attitudes to in-house water recycling in England and Wales. *Water and Environment Journal*, https://doi.org/10.1111/j.1747-6593.2002.tb00397.x.
16. Jenssen P. D., Heeb J., Huba-Mang E., Gnanakan K., Warner W. S., Refsgaard K., … Alsén, K. W. (2004). Ecological sanitation and reuse of wastewater ecosan. Ecosan, (March).
17. Koetse E. (2005). The implementation of DESAR concepts in two projects in Germany, (July). Retrieved from http://www.switchurbanwater.eu/outputs/pdfs/W4-1_GEN_PHD_Implementation_of_DESAR_concepts_in_two_projects_in_Germany_-_Koetse.pdf
18. Lamichhane K. M., Babcock R. W. (2013). Survey of attitudes and perceptions of urine-diverting toilets and human waste recycling in Hawaii. *Science of the Total Environment*, https://doi.org/10.1016/j.scitotenv.2012.11.039.
19. Libralato G., Volpi Ghirardini A., Avezzù F. (2012). To centralize or to decentralize: An overview of the most recent trends in wastewater treatment management. *Journal of Environmental Management*, https://doi.org/10.1016/j.jenvman.2011.07.010.
20. Lienert J., Larsen T. A. (2006). Considering user attitude in early development of environmentally friendly technology: A case study of NoMix toilets. *Environmental Science and Technology*, https://doi.org/10.1021/es060075o.
21. Lienert J., Larsen T. (2010). High acceptance of urine separation in seven European countries: a review. *Enviromental Science and Technology*, https://doi.org/10.1021/es9028765.
22. Mankad A. (2012). Decentralised water systems: Emotional influences on resource decision making. *Environment International*, https://doi.org/10.1016/j.envint.2012.01.002 .
23. Mankad A., Tapsuwan S. (2011). Review of socio-economic drivers of community acceptance and adoption of decentralised water systems. *Journal of Environmental Management*, https://doi.org/10.1016/j.jenvman.2010.10.037.
24. Mankad A., Tapsuwan S., Greenhill M. P., Malkin S. (2011). Motivational indicators of decentralised systems use among householders in South East Queensland. Urban Water Security Research Alliance Technical Report No. 44, (44).
25. Mankad A., Tucker D., Tapsuwan S., Greenhill M.P. (2010). Qualitative exploration of beliefs, values and knowledge associated with decentralised water supplies in South East Queensland Communities. Urban Water Security Research Alliance Technical Report No. 25. Social Analysis, (25).
26. Matsebe G., Osman A. (2012). Ecological sanitation in urban South Africa: Socio-cultural, design and operational challenges of Urine Diversion Dry (UDD) toilets and the impact on users' perceptions. 4th International Dry Toilet Conference, 1–13.
27. Mattila H. (2001). The role of public acceptance in the application of DESAR technology. In P. Lens, G. Zeeman, & G. Lettinga (Eds.), *Decentralised sanitation and reuse concepts, systems and implementation* (pp. 515-533). London, UK: IWA Publishing.
28. Moglia M., Alexander K. S., Sharma A. (2011). Discussion of the enabling environments for decentralised water systems. *Water Science and Technology*, https://doi.org/10.2166/wst.2011.443 .
29. Moglia M., Cook S., Sharma A. K., Burn S. (2010). Assessing decentralised water solutions: Towards a framework for adaptive learning. *Water Resources Management*, https://doi.org/10.1007/s11269-010-9696-7.

30. Moglia M., Sharma A. K. (2013). The need for monitoring the social performance of decentralised water systems. CSIRO: Water for a Healthy Country National Research Flagship.
31. Moglia M., Sharma A., Alexander K., Mankad A. (2011). Perceived performance of decentralised water systems: A survey approach. *Water Science and Technology: Water Supply*, https://doi.org/10.2166/ws.2011.081.
32. Nancarrow B. E., Porter N. B., Leviston Z. (2010). Predicting community acceptability of alternative urban water supply systems: A decision making model. Urban Water Journal, https://doi.org/10.1080/1573062X.2010.484500.
33. National Small Flows Clearinghouse. (2000). Decentralised wastewater treatment systems. *Pipeline, 11*(4), 1-8.
34. Nayono S., Lehn H., Kopfmüller J., Londong J. (2011). Options for decentralised waste water treatment in rural Karst Area in Gunung Kidul: Social acceptance. Asian Trans-Disciplinary Karst Conference, (January 7-10th), 1–11.
35. Pahl-Wostl C. (2005). Information, public empowerment, and the management of urban watersheds. *Environmental Modelling and Software*, 20(4 SPEC. ISS.), 457–467.
36. Parkinson J. (2003). Decentralised wastewater management in peri-urban areas in low-income countries. *Environment and Urbanization*, https://doi.org/10.1177/095624780301500119.
37. Pinkham R. D., Hurley E., Watkins K., Lovins A.B., Magliaro J., Etnier C., Nelson V. (2004). *Valuing Decentralized wastewater technologies: A catalog of benefits, costs, and economic analysis techniques*. USA: Rocky Mountain Institute, Snowmass, CO.
38. Pinkham R. D., Magliaro J., Kinsley M. (2004). Case Studies of Economic Analysis and Community Decision Making for Decentralized Wastewater Systems. Project No. WU-HT-02-03. Prepared for the National Decentralized Water Resources Capacity Development Project, Washington University, St. Louis, MO, by Rocky Mountain Institute, Snowmass, CO.
39. Poortvliet, P.M., Sanders, L., Weijma. J., & De Vries, J. R. (2018). Acceptance of new sanitation: The role of end-users' pro-environmental personal norms and risk and benefit perceptions. *Water Research, 131*, 90-99. https://doi.org/10.1016/j.watres.2017.12.032
40. Prouty, C., Mohebbi, S., & Zhang, Q. (2018). Socio-technical strategies and behavior change to increase the adoption and sustainability of wastewater resource recovery systems. *Water Research, 137*. 107-119. https://doi.org/10.1016/j.watres.2018.03.009
41. Quezada G., Walton A., Sharma A. (2016). Risks and tensions in water industry innovation: Understanding adoption of decentralised water systems from a socio-technical transitions perspective. *Journal of Cleaner Production*, https://doi.org/10.1016/j.jclepro.2015.11.018.
42. Roma E., Philp K., Buckley C., Xulu S., Scott D. (2013). User perceptions of urine diversion dehydration toilets: Experiences from a cross-sectional study in eThekwini municipality. *Water* SA, https://doi.org/10.4314/wsa.v39i2.15.
43. Ryan A. M., Spash C. L., Measham T. G. (2009). Socio-economic and psychological predictors of domestic greywater and rainwater collection: Evidence from Australia. *Journal of Hydrology*, https://doi.org/10.1016/j.jhydrol.2009.10.002.
44. Rygaard M., Godskesen B., Jørgensen C., Hoffmann B. (2014). Holistic assessment of a secondary water supply for a new development in Copenhagen, Denmark. *Science of the Total Environment*, https://doi.org/10.1016/j.scitotenv.2014.07.078.
45. Villarin, M.C., Merel, S. (2020). Paradigm shifts and current challenges in wastewater management. *Journal of Hazardous Materials, 390*, 122-139.

46. Wegelin-Schuringa M. (2001). Public awareness and mobilization for sanitation. In P. Lens, G. Zeeman, & G. Lettinga (Eds.), *Decentralised Sanitation and Reuse Concepts, Systems and Implementation* (pp. 534-551). London, UK: IWA Publishing.

Table S2. Description given to focus group participants about decentralized plants

Nowadays, wastewater treatment is mainly carried out in what are called centralized plants. These plants occupy an important surface area and receive wastewater from an entire city or a significant part of it. The so-called gray and black water from the homes and industries all go through the same collector until they reach the centralized plants. These mixed waters are treated in these plants through physical, chemical, and biological processes to eliminate contaminants.<br>As an alternative to this traditional system, the use of decentralized wastewater systems is being considered. Decentralized plants are much smaller in size, since they can be located in a building, urban area, or neighborhood, and receive only the wastewater from each of those places. With decentralized plants, the various types of water (gray and black) are collected separately and do not mix. In the decentralized plant, they are treated differently according to the characteristics of each. Additionally, this system allows reuse of the water once it is treated. While it cannot be used for drinking, the water can flush toilets, wash streets, or water gardens of the buildings, urban areas, and neighborhoods from which it is generated.
